# Cervical abscesses due to co-infection with *Burkholderia pseudomallei*, *Salmonella enterica* serovar Stanley and Mycobacterium *tuberculosis* in a patient with diabetes mellitus

**DOI:** 10.1186/1471-2334-13-527

**Published:** 2013-11-09

**Authors:** Helmi Sulaiman, Sasheela Ponnampalavanar, Kein Seong Mun, Claire M Italiano

**Affiliations:** 1 Division of Infectious Diseases, Department of Medicine, University Malaya Medical Centre, Kuala Lumpur, Malaysia; 2 Division of Anatomical Pathology, Department of Pathology, University Malaya Medical Centre, Kuala Lumpur, Malaysia

**Keywords:** Melioidosis, Tuberculosis, *Salmonella stanley*, Diabetes

## Abstract

**Background:**

Infections due to *Mycobacterium tuberculosis, Burkholderia pseudomallei* and non-typhoidal *Salmonella* cause significant morbidity and mortality throughout the world. These intracellular pathogens share some common predisposing factors and clinical features. Co-infection with two of these organisms has been reported previously but, to our knowledge, this is the first time that infection with all three has been reported in one person.

**Case presentation:**

In September 2010, a 58-year-old diabetic Malaysian male presented with fever and a fluctuant mass on the right side of his neck. *B. pseudomallei* was isolated from an aspirate of this lesion and there was radiological evidence of disseminated infection in the liver and spleen. The recurrence of clinical symptoms over ensuing months prompted further aspiration and biopsy of a cervical abscess and underlying lymph nodes. *Salmonella enterica* serovar Stanley and then *M. tuberculosis* were identified from these specimens by culture and molecular methods. The patient responded to targeted medical management of each of these infections.

**Conclusion:**

In endemic settings, a high index of suspicion and adequate tissue sampling are imperative in identifying these pathogenic organisms. Diabetes was identified as a predisposing factor in this case while our understanding of other potential risk factors is evolving.

## Background

Acquisition of infection requires the interplay between external factors, including environmental risk and exposure, and internal factors that contribute to host susceptibility*.* In tropical regions, such as Malaysia, organisms unique to the environment must always be considered in any infection. *Burkholderia pseudomallei, Mycobacterium tuberculosis* and *Salmonella stanley* are all intracellular organisms with the first two well-known for entering a latent phase in humans. A requirement for prolonged antibiotic treatment is also common to these three organisms. *M. tuberculosis,* one of the world’s leading infectious causes of death, has an estimated incidence in Malaysia of 81 per 100 000 population [1]. *B. pseudomallei* is a gram negative organism found in water and soil which is endemic to a number of South East Asian countries including Malaysia. Conversely, *S.* stanley is a food borne infection acquired by ingestion of contaminated food with a high incidence in neighbouring Thailand [2]. Herein, we present a case of a Malaysian diabetic male who had confirmed neck infection with these three organisms within a 6-month period.

## Case presentation

In September 2010, a 58-year-old male palm oil worker from Perak state, western Peninsular Malaysia, presented with a one-week history of fever and right-sided neck swelling. No other systemic symptoms were reported and he gave no history of weight loss. On admission to hospital, his temperature was 38°C and examination revealed a 5 cm × 4 cm mass on the right side of his neck with overlying erythema. 2 mL of pus was aspirated from this fluctuant mass prior to more definitive irrigation and debridement. Though the patient had no known co-morbidities his fasting blood glucose was found to be elevated at 9.9 mmol/L.

On day 2 of admission there was preliminary identification of *B. pseudomallei* from the aspirate. He was commenced on intravenous (IV) ceftazidime 2 g 3×/day and subcutaneous insulin for newly diagnosed diabetes mellitus. Computed tomography (CT) of the thorax and abdomen was performed which revealed micro-abscesses in the liver and spleen. These lesions were attributed to infection with *B. pseudomallei*. The patient completed a 2-week course of ceftazidime and was discharged on doxycycline 100 mg 2×/day and sulfamethoxazole/trimethoprim (cotrimoxazole) 400 mg/ 80 mg 2 tab 2×/day.

One month after discharge he was seen in clinic with a 10-day history of new left sided neck swelling. On examination, there was a 4 cm × 4 cm mass and fine needle aspirate (FNA) was performed. There was no growth from the specimen sent. The patient continued on doxycycline and co-trimoxazole with no improvement in the left sided-neck swelling.

Three months after initial presentation he was re-admitted and irrigation, debridement and biopsy of the neck abscess and left cervical lymph node was performed. While awaiting biopsy results he was commenced on intravenous imipenem and co-trimoxazole was increased to 4 tabs 2×/day. Doxycycline was ceased.

**Figure 1:**
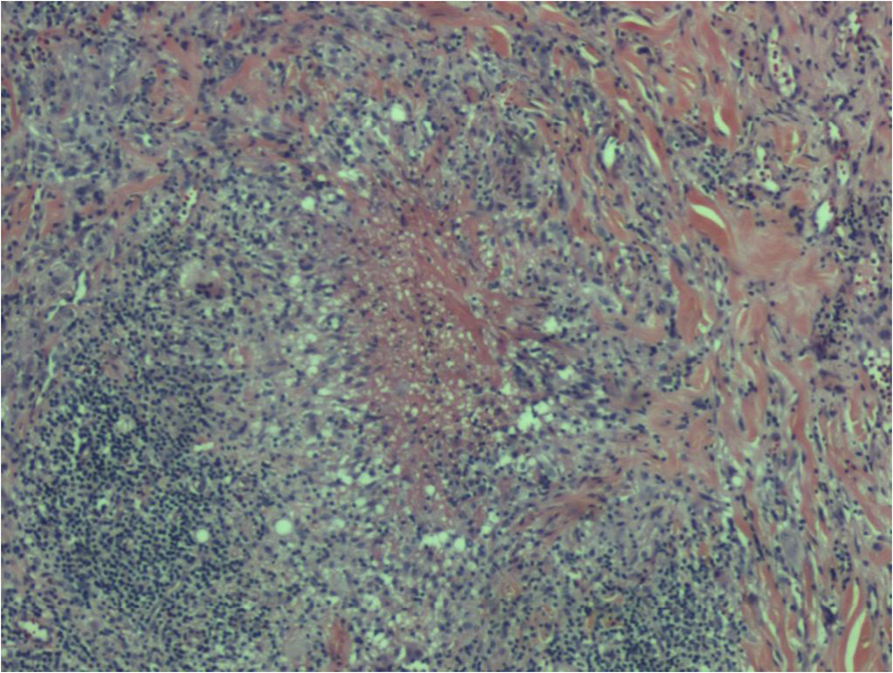
**Histopathological specimen of the neck.** This figure shows a necrotizing granuloma (Hematoxylin & Eosin, × 40 magnification).

**Figure 2:**
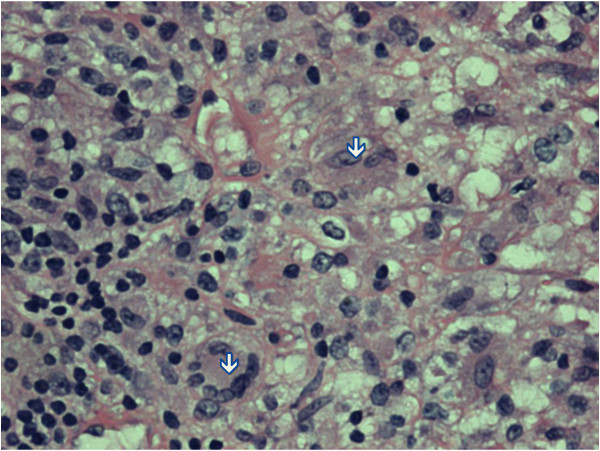
**Granuloma with epitheloid histiocytes.** This section is the high power view of the periphery of a granuloma showing epithelioid histiocytes [dirty white down arrow] and a Langhan’s giant cell [dirty white left arrow] (Hematoxylin & Eosin, × 200 magnification).

Biopsy of the neck abscess showed a partly ulcerated wedge of skin with pseudoepitheliomatous hyperplasia of the epidermis. The underlying dermis was infiltrated by aggregates of lymphoplasmacytic cells admixed with neutrophils and eosinophils, mostly congregating around the blood vessels and adnexae. In areas, necrotizing epithelioid granulomata (Figures 1 and 2) were seen. Langhan’s giant cells were also present. Histochemistry stains with Ziehl-Neelsen (ZN) and Gomori Methanamine Silver did not reveal any acid-fast bacilli or fungal organisms. The specimen from the lymph node was revealed to be a wedge of inflamed granulation tissue.

Unfortunately, despite the need for a full microbiological diagnosis, mycobacterial culture was not requested on this specimen. However, the pus and stool cultured *Salmonella* species. This was later identified as *Salmonella enterica* serovar Stanley via polymerase chain reaction (PCR) of the pus. The patient did give a history of eating imported nuts. As he had full dentures, poor oral hygiene was not thought to be a contributing factor to this infection. He received imipenem 500 mg 4×/day for 10 days followed by ciprofloxacin 750 mg 2×/day. He was discharged on this and co-trimoxazole was continued. Of note, trans-thoracic echocardiogram did not reveal any vegetation on the heart valves. CT of the neck, thorax and abdomen showed that the initial right-sided neck collection had resolved, while there was a new collection in the posterior region of the left side of the neck. In addition, the liver and splenic abscesses appeared unchanged. He was negative for human immunodeficiency virus (HIV) infection.

Despite treatment for the *S. stanley* infection, and continuation of high dose cotrimoxazole, there remained a residual left-sided neck collection. Antibiotics were ceased and a repeat FNA was performed a week after cessation of antibiotics to investigate for tuberculosis. When this did not identify an aetiological agent, a biopsy was performed which showed only acute on chronic inflammation. The ZN stain and mycobacterial culture were negative but PCR identified *M. tuberculosis*. The patient was commenced on isoniazid, rifampicin, pyrazinamide and ethambutol in combined tablet form to treat tuberculous lymphadenitis. His HBA1C was 7.1% on oral hypoglycaemic agents.

At review, after 1 month of treatment for tuberculosis, the left neck swelling had resolved but CT Abdomen did not show any change in the liver or splenic lesions. He completed 6 months of anti-tuberculosis treatment with no recurrence of symptoms but, despite treatment for *B. pseudomallei*, *S.* stanley and *M. tuberculosis,* there was no change in the liver or splenic lesions on repeated imaging.

At follow-up, more than 4 months after completion of all antibiotics, the patient was clinically well with no fevers or recurrence of neck swelling.

## Discussion

*M. tuberculosis*, *S. stanley*, and *B. pseudomallei* are all intracellular pathogens with the potential for latency in the body. Though co-infection with these organisms has been reported previously, infection with all three organisms in such a short time period and with occurrence in the neck has not. All organisms are endemic to the region and this patient’s work in a palm oil field is likely to have contributed to an exposure risk for *B. pseudomallei*. The patient’s previously undiagnosed diabetes was the only known co-morbidity likely to increase susceptibility to these infections. Other immunological factors have been shown to predispose to these three serious infections and it may be possible that this patient had other risk factors that we were not able to investigate for.

Dual infection with *M. tuberculosis* and *B. pseudomallei* has been reported previously including a case presenting with a neck abscess [3-5]. In the two cases where it was documented, both patients were diabetic as in this case. Though pneumonia is the commonest manifestation of infection in both diseases [6,7], infection of the neck and regional lymph nodes is also well described [8,9]. A case series of infections of the neck region secondary to *B. pseudomallei* has previously been reported from the same region of Malaysia as where this patient resides [9]. Importantly, both infections can present in a similar manner with protracted duration of symptoms and multi-organ involvement. In fact, *B. pseudomallei* infection can mimic *M. tuberculosis*, and vice-versa, especially in endemic regions [10].

*S. stanley* is a food borne disease in which outbreaks can occur in clusters following consumption of contaminated foods such as peanuts, alfalfa sprouts, fava beans, milk and cheese [11-13]. In neighbouring Thailand, the incidence of infection with this particular serovar is high, constituting the second commonest form of infection with *Salmonella* species [2].

Defects in the interleukin-12 (IL12) and interferon γ (IFNγ) pathway have been recognized as factors in co-infection with non-tuberculous mycobacteria and non-typhoidal salmonella [14]. IL-12 deficiency has been associated with concomitant persistent mycobacterial infection and disseminated salmonella infection [15]. IFN-γ deficiency and autoantibodies to IFN-γ have been linked to disseminated tuberculosis and non-tuberculous mycobacterial infection [16]. Additionally, autoantibodies to IFN-γ have been implicated in co-infection with non-tuberculous mycobacteria and other Burkholderia species [17].

Aside from these immunological defects, diabetes mellitus is a known risk factor for all three infections and is the commonest risk factor for infection with *B. pseudomallei* in studies from Australia, Thailand and Malaysia [6,18,19]. *B. pseudomallei* is a versatile organism that is able to employ a multitude of virulence factors in order to survive in an intracellular niche. Prior to entry into cells, it has been shown that this organism can interfere with complement activity and opsonisation by phagocytes [20]. Following its acquisition it may stay dormant until the host immunity falters and favors its multiplication. This may occur in the presence of diabetes, chronic alcoholism or renal impairment. It is possible that there is interplay between diabetes and other factors that predispose to infection with intracellular organisms. Reduction in interleukin-12 production in peripheral blood monocytic cells has been shown to increase a diabetic person’s susceptibility to melioidosis and tuberculosis. Unfortunately, further testing for immunological defects was not available at our institution and therefore predisposing causes other than diabetes could not be identified. In most developing and middle income countries, where these diseases are endemic, comprehensive immunological testing is not available and reinforces the need for a high degree of clinical suspicion.

*B. pseudomallei* and *M. tuberculosis* are endemic to Malaysia and the incidence of *S.* stanley infection in neighbouring Thailand is high. In this tropical environment, exposure to these organisms in this diabetic, and therefore susceptible patient, resulted in the unusual occurrence of three infections in the neck within a short time period. The diagnosis of *M. tuberculosis* must always be considered in unresolved infections and obtaining an adequate biopsy specimen to allow for identification of the organism is imperative.

## Conclusions

Both tuberculosis and melioidosis may masquerade as each other due to the nature of their signs and symptoms and multi-organ involvement. However, based on our experience with this patient, the presence of non-typhoidal salmonellosis may also complicate the clinical picture in diabetic subjects. This underscores the importance for clinicians to have a high index of suspicion and be aware of potential infections complicating diabetes, especially in the tropics, and hence have a systematic approach to investigating and managing such patients.

## Consent statement

Written informed consent was obtained from the patient for publication of this case report and any accompanying images. A copy of the written consent is available for review by the Editor of this journal.

## Competing interests

The authors declare that they have no competing interests.

## Authors’ contributions

Both CI and SP were involved in the management of the patient. The initial manuscript was prepared by CI. HS later contributed to the expansion of the manuscript, the analysis of the case and its discussion. KSM was involved in slide preparation, interpretation and preparation of the images of pathological specimens for the case report. All authors read and approved the final manuscript.

## Authors’ information

HS is an infectious diseases trainee in the Infectious Disease Unit at University Malaya Medical Centre. SP is a consultant in this unit while CI was a visiting consultant during this period. KSM is a pathologist and a lecturer in the pathology department of University Malaya.

## Pre-publication history

The pre-publication history for this paper can be accessed here:

http://www.biomedcentral.com/1471-2334/13/527/prepub
